# An Account of the Efficacy of the Acetate of Lead in Uterine Hæmorrhage

**Published:** 1808-01

**Authors:** George E. Mitchell

**Affiliations:** Elkton, Maryland


### *An Account of the Efficacy of the Acetate of Lead in Uterine Hæmorrhage.* By GEORGE E. MITCHELL, M. D. &c. *of Elkton, Maryland; communicated to* Dr. JACOB D. WACKER, *of Fredericksburg, Virginia.*

DEAR SIR,

AS experience is the only true way to obtain a perfect knowledge of the virtues of medicine, I feel disposed to inform you of my experience of the efficacy of acetate of lead, in several cases of uterine hæmorrhage, which have occurred in my practice, since I left the university of Pennsylvania. Although I was induced to believe it a valuable implement in the hands of a physician, in this form of disease, from the encomiums of professor Barton, yet my experience has greatly confirmed that belief; it has also convinced me that a student of the present day, may put more confidence in the recommendations of our professors, than many of the ancient writers are entitled to.

I have had five cases of this disease, in all which, this medicine was prescribed with evident success; in several of these cases blood-letting was used as it was indicated by the pulse. The acetate of lead was used according to the urgency of the case I generally commenced with one, two, or three grains, to be repeated every three or four hours, which always had the effect it was desired to produce. One case, which was alarming and obstinate, and which had been treated with the common remedies in this disease, will convince you of its superior efficacy.

A lady of delicate make, aged 29 years, was seized with uterine hæmorrhage; her physician had properly prescribed according to the state of her system; blood-letting had been used several times. Astringent powders of sulphate of alumine and gum kino had been given every hour; proper local applications had been used without much appa-

 rent rent advantage. The woman was much reduced, and she, as well as her physician, was sensible that her complaint, if not soon checked, would terminate her existence. I was consulted in her case, and recommended the acetate of lead: this practice was novel to the attending physician; but he agreed it should be tried: I prepared a dose of five grains, made into a bolus, with mucilage of gum arabic, and gave it her with a determination to repeat it, if it was found necessary; but in one hour it was evident the pill had produced the desired effect: the hæmorrhage was stopped, and did not return; next day she was ordered tonic powders of rubigo ferri, and columbo, which soon restored her to her former state of health.

Another case I think proper to relate to you. On the 25th of July last, I was attending a patient in the house of a gentleman in this neighbourhood, and whilst performing the operation of blood-letting, the lady of the family came into the room; she was in perfect health; but soon complained the sight of the blood made her sick; she hastened into another room, and was immediately taken with a profuse uterine hæmorrhage. I was called in, and as she was far advanced in pregnancy, and the hæmorrhage alarming, I concluded her in very imminent danger. As soon as possible, I gave her about five grains of this medicine, which very soon produced the wished for effect. Since which, at the proper time, she has born a healthy child. I have used it with success in active and passive hæmorrhage, without the assistance of any other remedy.

In the third case in which I have prescribed this medicine, I was alarmed to find my patient in a profuse ptyalism. I was accused of having used mercury, but conscious I had not given her any, could attribute it alone to this medicine. I recollected a woman in the Pennsylvania hospital, who was salivated by a few grains of the same medicine: if I remember aright, it was given by Dr. Rush in epilepsy. Dr. James Archer, of Hartford county, informed me yesterday, that the same circumstance had happened to a patient of his; he prescribed it for diarrhœa. I was not sensible of that disagreeable smell which is always the effect of mercury.

I have also used this medicine with success in alarming and obstinate cases of hæmoptysis. In none of the cases in which I used it, did any ill consequences follow, except the ptyalism produced in the third case. Indeed the experience of many, who have used this medicine in large and repeated doses, and the many mistakes * which have been made made with it with impunity, ought to dispel that improper timidity with which it is generally prescribed; and which often prevents its prescription, when it might be used with the most happy effects.

How valuable is that medicine with which the illustrious Rush and others, have cured epilepsy! Its efficacy (established by experience) externally and internally, in many forms of disease, is sufficient to convince the most sceptical that it is a very valuable medicine.

*February* 13, 1806.

